# Herpetic gingivostomatitis in a young adult

**DOI:** 10.11604/pamj.2022.41.113.32711

**Published:** 2022-02-08

**Authors:** Sho Katayama

**Affiliations:** 1 Department of Dermatology, Chitose City Hospital, Chitose, Japan

**Keywords:** Herpes simplex, herpetic gingivostomatitis, oral enanthema

## Image in medicine

An 18-year-old woman presented with a 7-day history of fever up to 39.2°C followed by painful oral enanthema. She had taken acetaminophen as a palliative medicine. Her past history was unremarkable. Physical examination showed hemorrhagic crusts and necrotic ulcers on the lips. Prominent swelling with dilated capillaries and white sloughs of the attached gingiva over the teeth were observed (A,B). Laboratory investigations were unremarkable, except for elevated C-reactive protein at 3.81 mg per deciliter (reference < 0.30). Serum anti-herpes simplex virus (HSV) IgM was positive, but IgG was negative. The Tzanck test was positive and polymerase chain reaction detected HSV type 1 (HSV-1) DNA. Primary HSV-1-induced herpetic gingivostomatitis were diagnosed. She was treated with oral valacyclovir, and the lesion completely resolved in 10 days. Classically, HSV-1 infection occurs during childhood through direct or indirect oral contact and develops herpetic gingivostomatitis as the primary lesion. In Africa, more than 90% of children have the HSV-1 antibody by 10 years of age. However, in socio-economically advanced countries, HSV-1 seroprevalence in children has been progressively declining and sexual transmission of HSV-1 has been increasing in adolescents and above, which can contribute to the adult onset herpetic gingivostomatitis.

**Figure 1:**
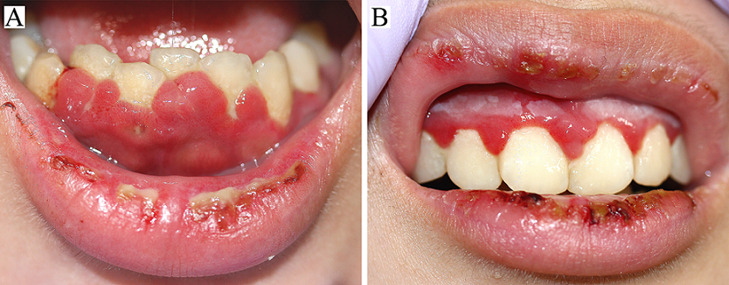
A) swelling with dilated capillaries, white sloughs, and shallow ulcers in the upper gums; B) prominent swelling with hemorrhages in the lower gums, and ulcers on the lower lip

